# SYT-SSX fusion genes and prognosis in synovial sarcoma

**DOI:** 10.1054/bjoc.2001.2088

**Published:** 2001-11

**Authors:** A Mezzelani, L Mariani, E Tamborini, V Agus, C Riva, S Lo Vullo, A Fabbri, M Stumbo, A Azzarelli, P G Casali, A Gronchi, G Sozzi, M A Pierotti, S Pilotti

**Affiliations:** Division of Anatomic Pathology and Cytology, Medical Statistics, Surgery of Soft Tissue Tumors, Medical Oncology A, Experimental Oncology, Istituto Nazionale per lo Studio e la Cura dei Tumori, Via Venezian 1, Milano, 20133, Italy

**Keywords:** synovial sarcoma, fusion transcripts, morphology, prognosis

## Abstract

A case series of 64 synovial sarcomas was characterized for the SYT-SSX fusion transcripts and statistically analysed in order to correlate molecular data with prognosis and morphology. SYT-SSX1 fusion transcript appeared to be an independent, though not reaching statistical significance (*P* = 0.183), prognostic factor clearly associated with a reduced metastasis-free survival. Regarding the association between transcript type and histologic subtype we found, a borderline *P* value (*P* = 0.067) between the SYT-SSX1 transcript and the biphasic subtype which, subsequently expanding the analysis to 70 cases, turned out to be significant. However, we could not confirm the prediction value of the biphasic subtype for the presence of the SYT-SSX1 transcript since in our hands 6 out 33 (18%) biphasic tumours carried the SYT-SSX2 transcript.© 2001 Cancer Research Campaign  http://www.bjcancer.com


British Journal of Cancer (2001) 85(10), 1535–1539
© 2001 Cancer Research Campaign
doi: 10.1054/ bjoc.2001.2088, available online at http://www.idealibrary.com on IDEAL
http://www.bjcancer.com

### SYT-SSX fusion genes and prognosis in synovial sarcoma

**A Mezzelani[1], L Mariani[2], E Tamborini[1], V Agus[1], C Riva[1], S Lo Vullo[2], A Fabbri[1], M Stumbo[1], A Azzarelli[3], PG Casali[4], A Gronchi[3], G Sozzi[5], MA Pierotti[5] and S Pilotti[1]**

[1]Division of Anatomic Pathology and Cytology; [2]Medical Statistics; [3]Surgery of Soft Tissue Tumors; [4]Medical Oncology A and [5]Experimental Oncology, Istituto Nazionale per lo Studio e la Cura dei Tumori, Via Venezian 1, 20133 Milano, Italy

**Summary** A case series of 64 synovial sarcomas was characterized for the SYT-SSX fusion transcripts and statistically analysed in order to correlate molecular data with prognosis and morphology. SYT-SSX1 fusion transcript appeared to be an independent, though not reaching statistical significance ($P = 0.183$), prognostic factor clearly associated with a reduced metastasis-free survival. Regarding the association between transcript type and histologic subtype we found, a borderline $P$ value ($P = 0.067$) between the SYT-SSX1 transcript and the biphasic subtype which, subsequently expanding the analysis to 70 cases, turned out to be significant. However, we could not confirm the prediction value of the biphasic subtype for the presence of the SYT-SSX1 transcript since in our hands 6 out 33 (18%) biphasic tumours carried the SYT-SSX2 transcript. © 2001 Cancer Research Campaign http://www.bjcancer.com

**Keywords**: synovial sarcoma; fusion transcripts; morphology; prognosis

Synovial Sarcoma (SS) is a distinct and fairly common kind of soft tissue sarcoma, occurring most often in young adults and having a predilection for the extremities (Enzinger and Weiss 2001). Two histologic subtypes are recognized: the biphasic, the most typical but less common sub-type, containing both an epithelial and a spindle cell component; and the monophasic subtype, entirely made up of spindle cells. A rare form of monophasic SS has been described, containing only epithelial-like cells (Meis-Kindblom et al, 1996).

Cytogenetically, SS is characterized by the translocation t(X;18)(p11.2;q11.2) in more than 95% of the cases. This translocation fuses the SYT gene from chromosome 18 with one of three closely related genes SSX1, SSX2 or SSX4, located at the X chromosome. The fused genes form a chimeric protein that seems to deregulate either the transcription or the expression of specific target genes. The current evidence suggests that this non-random SS translocation may be associated to the transactivation of a cascade of genes such as c-MET (Oda et al, 2000), c-KIT (Tamborini et al, 2001) and their ligands, and BCL-2 (Mancuso et al, 2000), yielding two different autocrine loops, one acting on the epithelial differentiation (c-MET), and the other working in an apoptosis-protecting program (BCL-2, c-KIT).

Synovial sarcoma has been traditionally regarded as a highly malignant tumour, but its prognosis varies greatly depending on a number of patients evaluated or clinico-pathologic features taken into consideration. One is tumour size, that has been shown to be an independent predictor of both distant recurrence and mortality (Lewis et al, 2000). Another is tumour grade. Recently, two grading systems have been proposed for SS. One is morphology-based and depends on the presence of atypia, necrosis and mitotic activity (Skytting et al, 1999). The second is clinico-pathologic oriented, and takes into account patient's age, tumour size, and the presence of a poorly differentiated tumour component. Both systems have been shown to split SSs into favourable and unfavourable cases (Bergh et al, 1999). Additional biological markers of possible prognostic significance have been recently investigated, including Ki-67 (Inagaki et al, 2000), c-myc (Shen et al, 2000), and the co-expression of HGF and c-MET (Oda et al, 2000).

Two recent studies have focused on the relevance of the different fusion gene types in affecting the outcome of disease (Kawai et al, 1998; Nilsson et al, 1999). Both studies showed a significant reduction of metastasis-free survival in patients carrying the SYT-SSXI fusion type of transcript when compared with those carrying the SYT-SSX2 fusion type. Furthermore, SYT-SSX1 and SYT-SSX2 fusion transcripts were shown to be significantly associated with biphasic and monophasic SSs, respectively.

Here we report on our own series of SS patients characterized for the presence and type of SYT-SSX fusion transcripts with the purpose of further verifying the value of the different SYT-SSX gene fusions as determinants of both prognosis and morphology in SS.

### MATERIALS AND METHODS

A total of 72 consecutive cases of SS collected between 1989 and 1999 in which frozen tissue samples were available were retrieved. The criteria applied for the diagnosis of biphasic and monophasic type were those reported by Enzinger and Weiss (2001) i.e., the presence of epithelial and spindle cell components in varying proportions in the former and a fibrosarcoma-like spindle cell component with no demonstrable epithelial component in the latter. Six cases were excluded from the study for the following reasons: four patients already had metastatic disease at the time of diagnosis, and therefore they could not be assessed for time to occurrence of distant metastases (the primary study endpoint); one

*Received 11 April 2001*
*Revised 23 July 2001*
*Accepted 7 August 2001*

*Correspondence to:* S Pilotti patient did not receive radical surgery; and another patient had previously a Ewing sarcoma/pPNET of the thoracic region, carrying the t(11;22)(q24;q12) translocation.

The tissue samples analysed molecularly were from primary tumours in 26 patients, from recurrences in 18, and from distant metastases in 20 cases.

The treatment of the patients affected by localized tumours (primary in 26 and recurrent in 18) consisted of conservative surgery in 41, (associated to radiation therapy whenever indicated), and major disarticulation in the other 3. Systemic chemotherapy was administered according to standard protocols, with 31 patients treated by adjuvant or neoadjuvant therapy.

All patients with distant metastases to lung (20 cases) had at least one excision of the pulmonary mass(es), and 9 of them underwent systemic chemotherapy, as well as 22 of the remaining patients.

### Molecular analysis

A frozen tumour fragment from each case was mechanically disaggregated and total RNA was isolated using the RNAzoIB extraction system (TEL-TEST, Friendswood, Texas). One μg of total RNA was reverse-transcribed into cDNA using oligo(dt) primers and reverse transcriptase (Superscript, Gibco BRL, Paisley, UK), according to the manufacturer's recommendations. The integrity of cDNA was tested by the amplification of the ubiquitous gene β-actine (Adams et al, 1995). The detection of the putative SYT-SSX1 and SYT-SSX2 fusion gene was carried out with the primers SYT 5′CAA CAG CAA GAT GCA TAC CA3′, SSX1 5′GGT GCA GTT GTT TCC CAT CG3′ and SSX2 5′GGC ACA GCT CTT TCC CAT CA3′ (Kawai et al, 1998). PCR conditions were the following: 35 cycles of denaturation at 94˚C for 30 sec, annealing at 58˚C for 1 min, and elongation at 72˚C for 1 min. The detection of the putative SYT-SSX4 fusion transcript was carried out by a nested RT-PCR with the following primers: SYT external 5′CAA CAG CAA GAT GCA TAC CA3′ and SSX external 5′TGC TAT GCA CCT GAT GAC GA3′ in the first step and SYT internal 5′AGA CCA ACA CAG CCT GGA CCA3′ and SSX4 5′GGC ACA GCT GTT TCC CAT CA3′ in the second step (Skytting et al, 1999). The thermal profile for both reactions was identical to that mentioned for the detection of SYT-SSX1 and SYT-SSX2, except that the annealing temperature in the first step was 52˚C. Each reaction included cDNA from normal mesenchymal tissue as negative control. The amplification products of all the PCR reactions were analysed on 2% agarose gel that can clearly detect both the classic SYT-SSX fusion trancripts and all the described variants (Fligman et al, 1995; Safar et al, 1998)

All the sequence reactions were carried out using an automated sequencing system (377 DNA Sequencer, ABI PRISM PE, Applied Biosystem) following standard protocols.

### Statistical methods

The association of SYT-SSX gene transcripts (SYT-SSX1 and SYT-SSX2) with the various clinical and pathologic parameters investigated was tested using the Pearson chi-square test, with exact P computation (SAS Institute Inc., 1996).

The main endpoint of the study was metastasis-free survival. Time to distant metastasis was computed from the date of disease diagnosis to the date of event occurrence, or the date of last follow-up visit for patients free from distant metastases. Metastasis-free survival curves were obtained with the Kaplan-Meier method. The prognostic value of SSX gene transcripts on metastasis-free survival was assessed using the Cox model, both without and with adjustment for the following covariates: age (< 25 versus ≥ 25 years); tumour site (head and trunk versus limbs) tumour size (> 5 versus ≤ 5 cm); and tumour histologic subtype, (monophasic versus biphasic). The choice of covariates and their categorization was done before the analysis and relied on clinical expertise and literature data. Patients for which the source of tumour samples was a recurrent lesion represent a selected population, in that they entered the study cohort given that they had developed such a recurrence. Their behaviour was expected to differ from that of patients with samples from the primary tumour. Exploration of our data clearly showed that subjects with samples from distant metastases differed prognostically from the remaining ones. For this reason Cox model analyses were stratified according to the source of the tumour sample as follows: local sample cohort, encompassing primary tumour and/or local recurrences; and metastatic sample cohort. The choice of covariates and their categorization was done before the analysis and relied on clinical expertise and literature data.

Patients who had the assessment performed on both types of tissue (6 cases) were included in the local sample cohort. In all such cases, the SYT-SSX gene translocation results yielded always concordant results in the different samples.

The covariates were entered into the Cox model by means of 0–1 indicator variables. We also tested the interaction terms between SYT-SSX gene transcripts and the type of tumour sample analysed or the histologic subtype. The proportional hazard assumption implied by the model was checked using log-minus log survival plots. Hazard ratio (HR) estimates obtained with the Cox model were reported together with the corresponding 95% confidence limits and associated *P*-value. For a given covariate, a hazard ratio equal to one denotes a lack of prognostic effect, whereas values above (below) one denote an increased (decreased) risk of occurrence of the event in a particular category, compared to the reference category.

Analyses were carried out using SAS™ (SAS Institute Inc., 1990). *P*-values reported are two-sided; a *P*-value below 0.05 was considered significant, whereas a *P*-value between 0.10 and 0.05 was considered borderline.

### RESULTS

### Molecular analysis

Among the 72 patients with SS (42 monophasic and 30 biphasic) analysed for the SYT-SSX fusion genes, the SYT-SSX1 and the SYT-SSX2 fusion types were detected in 44 (61.1%) and 26 (36.1%) of the tumours, respectively. The remaining 2 (2.8%) cases had an SYT-SSX4 fusion transcript. 20 of the 42 (47.6%) monophasic tumours showed a SYT-SSX1 fusion type, 20 (47.6%) a SYT-SSX2, and 2 (4.8%) a SYT-SSX4. Conversely, 24 (80%) of the 30 biphasic SSs showed a SYT-SSX1, and 6 (20%) a SYT-SSX2 fusion type.

By amplifying the DNA across the breakpoint region no electrophoretically different bands were detected. The PCR product of two cases were sequenced and used as positive control for SYT-SSX1 and SYT-SSX2 fusion transcripts, respectively. The PCR products of the two SYT-SSX4 cases were also sequenced, as well as those of six biphasic SS showing an SYT-SSX2 fusion transcript.

 *© 2001 Cancer Research Campaign*

### Statistical analysis

The two cases carrying the SYT-SSX4 fusion transcript were discarded. Since six cases had already been eliminated (see Patients and Methods), the statistical analysis was performed on 64 cases, 40 SYT-SSX1 and 24 SYT-SSX2, diagnosed between 1989 and 1999, all of which had loco-regional disease at presentation.

The prevalence of the SYT-SSX1 transcript (Table 1) was higher among males then females, in tumours ≤ 5 cm in size, and in biphasic lesions. Tumour site did not appear to substantially affect the prevalence of the SYT-SSX1 transcript, and there was no evident trend with patient age. A borderline *P* was obtained for histologic subtype ($P = 0.067$).

Forty-five distant metastases were recorded. Metastasis-free survival curves according to the type of transcript were substantially overlapping in local sample cohort (Figure 1). On average, metastasis-free survival was about 50% at 5 years. In the metastatic sample cohort, the survival tended to be lower for SYT-SSX1 carrying tumours (Figure 2).

The unadjusted hazard ratio estimate for SYT-SSX (SYT-SSX1 versus SYT-SSX2) was 1.35 (95% confidence limits: 0.71–2.57; $P = 0.365$). Results of the multivariable Cox regression model are reported in Table 2. The hazard ratio estimate adjusted for age, tumour site, tumour size and histologic sub-type was 1.61 (0.80–3.23; $P = 0.183$) overall. The figures obtained according to the type of tissue sample analysed or the histologic subtype were: 1.37 (0.55–3.38) for the local sample cohort, 1.95 (0.71–5.37) for the metastatic sample cohort ($P = 0.595$ for the interaction between SYT-SSX and type of sample analysed); 1.36 (0.62–3.01) for monophasic tumours, 2.59 (0.67–10.0) for biphasic tumours ($P = 0.406$ for the interaction between SYT-SSX and histologic subtype). Regarding the remaining covariates, a less favourable prognosis (hazard ratio > 1) was associated with age < 25 years, head/trunk tumour location, tumour size > 5 cm, and monophasic histologic subtype. However, none of these differences was significant on statistical analysis.

**Table 1** Prevalence of SSX1 transcripts according to main patient and disease characteristics

| | No. of cases | SSX1 | % | *P* |
|---|---|---|---|---|
| Sex | | | | 0.118 |
| Female | 36 | 19 | 52.8 | |
| Male | 28 | 21 | 75.0 | |
| Age (years) | | | | 0.115 |
| ≤ 20 | 12 | 7 | 58.3 | |
| 21–30 | 16 | 11 | 68.8 | |
| 31–40 | 12 | 4 | 33.3 | |
| 41–50 | 15 | 10 | 66.7 | |
| > 50 | 9 | 8 | 88.9 | |
| Tumour site | | | | 1.000 |
| Limbs | 44 | 28 | 63.6 | |
| Head/trunk | 20 | 12 | 60.0 | |
| Tumour size (cm) | | | | 0.196 |
| NR | 14 | 7 | 50.0 | |
| ≤ 5 | 19 | 15 | 79.0 | |
| > 5 | 31 | 18 | 58.1 | |
| Histologic sub-type | | | | 0.067 |
| Monophasic | 38 | 20 | 52.6 | |
| Biphasic | 26 | 20 | 76.9 | |

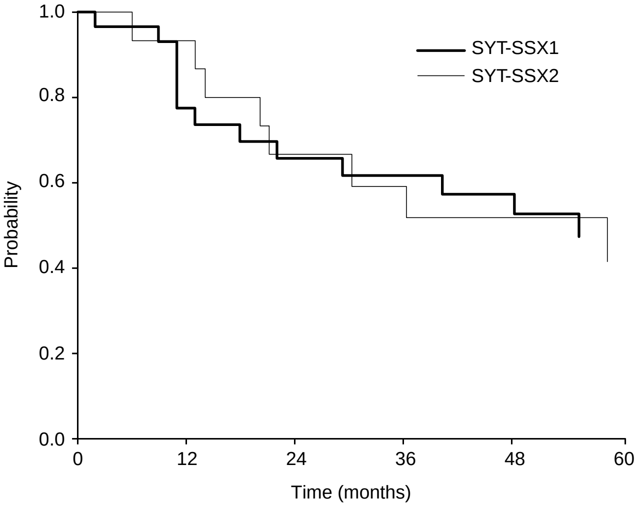

**Figure 1** Metastasis-free survival curves according to the type of transcript in the local sample cohort

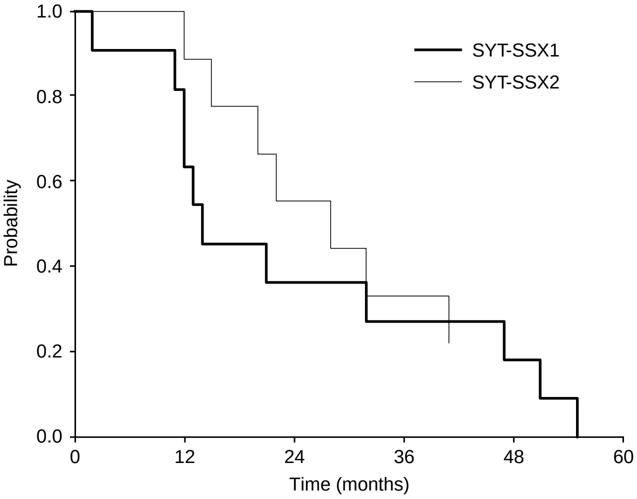

**Figure 2** Metastasis-free survival curves according to the type of transcript in the metastatic sample cohort

### DISCUSSION

The present study was undertaken to further evaluate the predictive value of the two alternative forms of the SYT-SSX fusion transcript (SYT-SSX1 and SYT-SSX2), concerning the prognosis and the morphology of SS.

The statistically valuable case material consisted of 64 cases where samples were obtained from 44 local tumours (26 primary and 18 local recurrent tumours) and 20 metastases. The tumours were classified as 38 monophasic and 26 biphasic and characterized as 40 with SYT-SSX1 and 24 with SYT-SSX2. PCR amplification and the subsequent electrophoretical analysis of the obtained products revealed band size compatible with the presence of the usual SYT-SSX genes fusion types in all the cases. For this reason after having verified the sequence of control cases we did not perform the DNA sequencing of all the PCR products, in the light also of Nilsson et al data which did not find uncommon rearrangements in fusion transcripts of the same size (Nilsson et al, 1999).

*© 2001 Cancer Research Campaign*

**Table 2** Results of the multivariate Cox model: hazard ratio estimates (HR), corresponding 95% confidence limits (CL) and *P*-value

| Variable | Reference category | HR | 95% CL | *P* |
|---|---|---|---|---|
| Fusion transcript | | | | |
| SSX1 | SSX2 | 1.61 | 0.80–3.23 | 0.183 |
| Age (years) | | | | |
| < 25 | ≥ 25 | 1.11 | 0.53–2.30 | 0.787 |
| Tumour site | | | | |
| Head/trunk | Limbs | 1.50 | 0.73–3.07 | 0.267 |
| Tumour size (cm) | | | | |
| > 5 | ≤ 5 | 1.89 | 0.85–4.19 | 0.119 |
| Histologic sub-type | | | | |
| Monophasic | Biphasic | 1.41 | 0.68–2.96 | 0.359 |

According to the same authors a tumour gene profile assessed on samples from patients enrolled at the time of primary/recurrent tumours may differ from that performed on samples from patients with metastatic disease (Nilsson et al, 1999). By contrast, the translocation (X;18)(p11.2;q11.2) is a non-random genetic hallmark stable over the disease progression. Thus, after a preliminary analysis performed in our series showing that cases with primary tumour or local recurrence samples (local sample cohort) had similar outcome in terms of metastasis-free survival, we split our case material into local and metastatic sample cohorts. In such a way we accounted for the heterogeneity in the tumour material used, at variance with previous work done by Kawai et al (1998). There was also some evidence of a possible imbalance in the distribution of a known prognostic characteristic such as tumour size between SYT-SSX1 and SYT-SSX2 cases (Table 1). Cox model multivariable analysis was aimed at removing possible confounding due to such an association.

As estimated by hazard ratios (HR) of the multivariate Cox regression model, tumour size turned out to be the factor more strongly associated with poor prognosis (HR = 1.89), followed by the type of SYT-SSX fusion (HR = 1.61). When investigating the effect of the different SYT-SSX transcripts according to the type of sample and histologic subtype, we observed a higher HR in the group of the metastatic sample cohort (HR 1.95) than in the local sample cohort (HR 1.36), and in biphasic tumours (HR 1.95) than in monophasic ones (HR 1.36). All together, the data were consistent with a clear, though not significant, less favourable prognostic effect of the SYT-SSX1 fusion type in metastatic sample cohort and biphasic tumours.

As compared with previous findings our HR estimates were clearly lower. In particular the findings observed by multivariate analysis were 3.0 (95% confidence limits, 1.1–0.80, *P* = 0.003) in the first series published (Kawai et al, 1998) and 7.4 (1.5–36.0, *P* = 0.004) in the second one (Nilsson et al, 1999). Notably, the latter considered only patients with SYT-SSX fusion transcripts assessed on primary tumours, whereas in the former, the fusion transcript assessment was performed not only on primary tumour, but also on local recurrence and metastasis.

Regarding tumour size, our results confirm also the well-established (Brodsky et al, 1992) and recently-reconfirmed evidence (Lewis et al, 2000; Machen et al, 1999) of an association between large tumour size (> 5 cm) and poor clinical outcome. Among the remaining variables considered, the age < 25 years, the axial tumour location and the monophasic pattern correlated, though not significantly, with a less favourable outcome in keeping again with the literature (Bergh et al, 1999).

The analysis of association between fusion transcripts and clinico-pathological variables showed, in addition to a higher prevalence of SYT-SSX1 in males, and in tumour smaller than 5 cm in diameter, an association, with a borderline *P* value (*P* = 0.067), between such a fusion transcript and the biphasic SS subtype. We observed in fact six (23%) biphasic SSs carrying a sequence-verified SYT-SSX2 fusion transcript. Interestingly three out of six belonged to children, and involved uncommon sites. It is worth mentioning that the Fisher's exact test performed on the entire series of 70 cases (see Materials and Methods), yielded a significant result (*P* = 0.010) (Mezzelani et al, 2000), in keeping with recent reports (Kawai et al, 1998; Antonescu et al, 2000).

In conclusion we reconfirm the association between the type of SYT-SSX fusion transcript and histologic sub-type. Regarding the clinical outcome, our data show a trend, not reaching statistical significance, of the SYT-SSX fusion transcript as an independent prognostic factor coherent with previous findings. However, in consideration of the heterogeneous effects estimated for this factor in currently available studies, we think that the prognostic effect of SYT-SSX fusion transcript deserves further confirmation on larger patient groups.

### ACKNOWLEDGEMENT

This work was supported by grants from 'Ministero della Sanità. Ricerca Finalizzata 1998-ICS030. 1/RF98.32-Italy' and by AIRC (Associazione Italiana per la Ricerca sul Cancro), grant n. 420.198.122. The authors are grateful to Dr Juan Rosai for the critical revision of the manuscript.

### REFERENCES

Adams V, Kempf VW, Hassams S and Briner J (1995) Determination of hexokinase isoenzyme I and II composition by RT-PCR: Increased hexokinase isoenzima II in human renal cell carcinoma. *Biochem Mol Med* **54**: 53–58

Antonescu CR, Kawai A, Leung DH, Lonardo F, Woodruff JM, Healey JH and Ladanyi M (2000) Strong association of SYT-SSX fusion type and morphologic epithelial differentiation in synovial sarcoma. *Diagn Mol Pathol* **9**: 1–8

Bergh P, Meis-Kindblom JM, Gherlinzoni F, Berlin O, Bacchini P, Bertoni F, Gunterberg B and Kindblom LG (1999) Synovial Sarcoma. Identification of low grade and high risk groups. *Cancer* **85**: 2596–2607

Brodsky JT, Burt ME, Hajdu SI, Casper ES and Brennan MF (1992) Tendosynovial sarcoma: clinicopathologic features, treatment and prognosis. *Cancer* **70**: 484–489

Enzinger FM and Weiss SW (2001) Chapter 37 In: *Soft Tissue Tumors*, 4 edn. Mosby: St Louis MO

Fligman I, Lonardo F, Jhanwar SC, Gerald WL, Woodruff J and Ladanyi M (1995) Molecular diagnosis of Synovial Sarcoma Characterization of a variant SYT-SSX2 fusion transcript. *Am J Pathol* **147**: 1592–1599

Inagaki H, Nagasaka T, Otsuka T, Suihura E, Nakashima N and Eimoto T (2000) Association of SYT-SSX fusion types with proliferative activity and prognosis in Synovial Sarcoma. *Mod Pathol* **13**: 482–488

Kawai A, Woodruff J, Healey JH, Brennan MF, Antonescu CR and Ladanyi M (1998). SYT-SSX gene fusion as a determinant of morphology and prognosis in synovial sarcoma. *N Engl J Med* **338**: 153–160

Lewis JJ, Antonescu CR, Leung DHY, Blumberg D, Healey JH, Woodruff JM and Brennan MF (2000) Synovial Sarcoma: a multivariate analysis of prognostic factors in 112 patients with preliminary localized tumors of the extremity. *J Clin Oncol* **18**: 2087–2094

Machen SK, Easley KA and Goldblum JR (1999) Synovial Sarcoma of the extremities. A clonicopathologic study of 34 cases, icluding semi-quantitative analysis of spindled, epithelial and poorly differentiated areas. *Am J Surg Pathol* **23**: 268–275

Mancuso T, Mezzelani A, Riva C, Fabbri A, Dal Bo L, Sampietro G, Perego P, Casali P, Zunino F, Sozzi G, Pierotti MA and Pilotti S (2000) Analysis of SYT-SSX fusion transcript and bcl2 expression and phosphorylation status in synovial sarcoma. *Lab Invest* **80**: 805–813

Meis-Kindblom JM, Stenman G and Kindblom L-G (1996) Differential diagnosis of small round cell tumors. *Semin Diagn Pathol* **13**: 213–241

*© 2001 Cancer Research Campaign*

Mezzelani A, Dagrada GP, Sozzi G, Pierotti MA and Pilotti S (2000) SYT-SSX fusion transcripts and epithelial differentiation in synovial sarcoma. *Diagn Mol Pathol* **9**: 234–235, (letter)

Nilsson G, Skytting B, Xie Y, Brodin B, Perfeckt R, Mandahal N, Lundemberg J, Uhlen M and Larsson O (1999) The SYT-SSX1 variant of synovial sarcoma is associated with a high rate of tumor cell proliferation and poor clinical outcome. *Cancer Research* **59**: 3180–3184

Oda Y, Sakamoto A, Saito T, Kinukawa N, Iwamoto Y and Tsuneyoshi M (2000) Expression of hepatocyte growth factor (HGF)/Scatter factor and its receptor c-Met correlates with poor prognosis in Synovial Sarcoma. *Hum Pathol* **31**: 185–192

Safar A, Wickert R, Nelson M, Neff JR and Bridge JA (1998) Characterization of a variant SYT-SSX1 Synovial Sarcoma fusion transcript. *Diagn Mol Pathol* **7**: 283–287

SAS Intitute Inc. 1990 *SAS/STAT™ User's Guide, Version 6*, Fourth Edition, Volumes 1 and 2, Cary NC: SAS Institute Inc. USA

SAS Institute Inc 1996 *SAS/STAT software: Changes and enhancements for release 6.12*. Cary, NC, SAS Institute Inc, pp. 15–17.

Shen J, Scotlandi K, Baldini N, Manara MC, Benini S, Cerisano V, Picci P and Serra M (2000) Prognostic significance of nuclear accumulation of c-myc and mdm2 proteins in Synovial Sarcoma of the extremities. *Oncology* **58**: 253–260

Skytting B, Meis-Kindblom JM, Larsson O, Virolainen M, Perfekt R, Akerman M and Kindblom LG (1999) Synovial Sarcoma: identification of favorable and unfavorable histologic types: a Scandinavian sarcoma group study of 104 cases. *Acta Orthop Scand* **70**: 534–554

Skytting B, Nillson G, Brodin B, Xie Y, Lundemberg J, Uhlen M and Larsson O (1999) A novel fusion gene, SYT-SSX4 in synovial sarcoma. *J Natl Cancer Inst* **91**: 974–975

Tamborini E, Papini D, Mezzelani A, Riva C, Azzareli A, Sozzi G, Pierotti MA and Pilotti S (2001) c-KIT and c-KIT ligand (SCF) in synovial sarcoma (SS): a mRNA expression analysis in 23 cases. *BJC* **85**: 405–411

*© 2001 Cancer Research Campaign*

## References

[PDF_00400] Adams V., Kempf W., Hassam S., Briner J. (1995). Determination of hexokinase isoenzyme I and II composition by RT-PCR: increased hexokinase isoenzyme II in human renal cell carcinoma.. Biochem Mol Med.

[PDF_00403] Antonescu C. R., Kawai A., Leung D. H., Lonardo F., Woodruff J. M., Healey J. H., Ladanyi M. (2000). Strong association of SYT-SSX fusion type and morphologic epithelial differentiation in synovial sarcoma.. Diagn Mol Pathol.

[PDF_00406] Bergh P., Meis-Kindblom J. M., Gherlinzoni F., Berlin O., Bacchini P., Bertoni F., Gunterberg B., Kindblom L. G. (1999). Synovial sarcoma: identification of low and high risk groups.. Cancer.

[PDF_00409] Brodsky J. T., Burt M. E., Hajdu S. I., Casper E. S., Brennan M. F. (1992). Tendosynovial sarcoma. Clinicopathologic features, treatment, and prognosis.. Cancer.

[PDF_00413] Fligman I., Lonardo F., Jhanwar S. C., Gerald W. L., Woodruff J., Ladanyi M. (1995). Molecular diagnosis of synovial sarcoma and characterization of a variant SYT-SSX2 fusion transcript.. Am J Pathol.

[PDF_00416] Inagaki H., Nagasaka T., Otsuka T., Sugiura E., Nakashima N., Eimoto T. (2000). Association of SYT-SSX fusion types with proliferative activity and prognosis in synovial sarcoma.. Mod Pathol.

[PDF_00419] Kawai A., Woodruff J., Healey J. H., Brennan M. F., Antonescu C. R., Ladanyi M. (1998). SYT-SSX gene fusion as a determinant of morphology and prognosis in synovial sarcoma.. N Engl J Med.

[PDF_00422] Lewis J. J., Antonescu C. R., Leung D. H., Blumberg D., Healey J. H., Woodruff J. M., Brennan M. F. (2000). Synovial sarcoma: a multivariate analysis of prognostic factors in 112 patients with primary localized tumors of the extremity.. J Clin Oncol.

[PDF_00426] Machen S. K., Easley K. A., Goldblum J. R. (1999). Synovial sarcoma of the extremities: a clinicopathologic study of 34 cases, including semi-quantitative analysis of spindled, epithelial, and poorly differentiated areas.. Am J Surg Pathol.

[PDF_00430] Mancuso T., Mezzelani A., Riva C., Fabbri A., Dal Bo L., Sampietro G., Perego P., Casali P., Zunino F., Sozzi G. (2000). Analysis of SYT-SSX fusion transcripts and bcl-2 expression and phosphorylation status in synovial sarcoma.. Lab Invest.

[PDF_00434] Meis-Kindblom J. M., Stenman G., Kindblom L. G. (1996). Differential diagnosis of small round cell tumors.. Semin Diagn Pathol.

[PDF_00452] Mezzelani A., Dagrada G. P., Sozzi G., Pierotti M. A., Pilotti S. (2000). SYT-SSX fusion transcripts and epithelial differentiation in synovial sarcoma.. Diagn Mol Pathol.

[PDF_00455] Nilsson G., Skytting B., Xie Y., Brodin B., Perfekt R., Mandahl N., Lundeberg J., Uhlén M., Larsson O. (1999). The SYT-SSX1 variant of synovial sarcoma is associated with a high rate of tumor cell proliferation and poor clinical outcome.. Cancer Res.

[PDF_00459] Oda Y., Sakamoto A., Saito T., Kinukawa N., Iwamoto Y., Tsuneyoshi M. (2000). Expression of hepatocyte growth factor (HGF)/scatter factor and its receptor c-MET correlates with poor prognosis in synovial sarcoma.. Hum Pathol.

[PDF_00463] Safar A., Wickert R., Nelson M., Neff J. R., Bridge J. A. (1998). Characterization of a variant SYT-SSX1 synovial sarcoma fusion transcript.. Diagn Mol Pathol.

[PDF_00470] Shen J., Scotlandi K., Baldini N., Manara M. C., Benini S., Cerisano V., Picci P., Serra M. (2000). Prognostic significance of nuclear accumulation of c-myc and mdm2 proteins in synovial sarcoma of the extremities.. Oncology.

[PDF_00474] Skytting B., Meis-Kindblom J. M., Larsson O., Virolainen M., Perfekt R., Akerman M., Kindblom L. G. (1999). Synovial sarcoma--identification of favorable and unfavorable histologic types: a Scandinavian sarcoma group study of 104 cases.. Acta Orthop Scand.

[PDF_00478] Skytting B., Nilsson G., Brodin B., Xie Y., Lundeberg J., Uhlén M., Larsson O. (1999). A novel fusion gene, SYT-SSX4, in synovial sarcoma.. J Natl Cancer Inst.

[PDF_00481] Tamborini E., Papini D., Mezzelani A., Riva C., Azzarelli A., Sozzi G., Pierotti M. A., Pilotti S. (2001). c-KIT and c-KIT ligand (SCF) in synovial sarcoma (SS): an mRNA expression analysis in 23 cases.. Br J Cancer.

